# Improvement in Quality of Life with Use of Ambient-Assisted Living: Clinical Trial with Older Persons in the Chilean Population

**DOI:** 10.3390/s23010268

**Published:** 2022-12-27

**Authors:** Carla Taramasco, Carla Rimassa, Felipe Martinez

**Affiliations:** 1 Instituto de Tecnología para la Innovación en Salud y Bienestar, Facultad de Ingeniería, Universidad Andrés Bello, Quillota 980, Viña del Mar 2531015, Chile; 2 Millennium Nucleus of Sociomedicine, Santiago 8320000, Chile; 3 Escuela de Fonoaudiología, Interdisciplinary Center for Territorial Health Research (CIISTe), Facultad de Medicina, Campus San Felipe, Universidad de Valparaíso, La Troya/El Convento S/N, San Felipe 2170000, Chile; 4 Facultad de Medicina, Escuela de Medicina, Universidad Andrés Bello, Viña del Mar 2531015, Chile; 5 Concentra Investigación y Educación Biomédica, Viña del Mar 2531015, Chile

**Keywords:** ambient assisting living, quality of life, healthy aging, sensors, age-friendly environments, age in place

## Abstract

In Chile, 18% of the population is over 60 years old and is projected to reach 31% in three decades. An aging population demands the development of strategies to improve quality of life (QoL). In this randomized trial, we present the implementation and evaluation of the Quida platform, which consists of a network of unintrusive sensors installed in the houses of elderly participants to monitor their activities and provide assistance. Sixty-nine elderly participants were included. A significant increase in overall QoL was observed amongst participants allocated to the interventional arm (*p* < 0.02). While some studies point out difficulties monitoring users at home, Quida demonstrates that it is possible to detect presence and movement to identify patterns of behavior in the sample studied, allowing us to visualize the behavior of older adults at different time intervals to support their medical evaluation.

## 1. Introduction

The world’s population is progressively aging [1]. Currently, in Chile, 18% of the country’s population is 60 years or older [2], with the regions of Valparaíso (12.5%) and Metropolitan (36.5%) [3] accounting for the highest proportions. This proportion is expected to increase nationwide, and it has been projected that in three decades, the group of older people will correspond to 31% of the entire population, thus making Chile the second-highest country in terms of the population of older people in Latin America [4]. These figures are relevant when looking at data from the Economic Commission for Latin America and the Caribbean (CEPAL), whose estimates indicate that by 2030 worldwide, the percentage of this age group will reach 16.4% [5]. That is, this proportion in Chile is already exceeded, which drives the diversification of the strategies that are oriented to generate protected environments, which make it possible to improve quality of life (QoL), providing safe environments that give well-being to the elderly.

To attain this objective, it should be kept in mind that aging is associated with the development of several diseases that gradually deteriorate physical and mental health, causing functional limitations, disability, and dependence [5]. It is known that 80% of the disease burden in adults over 65 corresponds to chronic conditions [6]. Chronic and neurodegenerative diseases generate a decrease in motor skills, preventing older people, to varying degrees, from performing some activities, including using the telephone, shopping, cooking, using transportation, taking medicines, and handling money [7]. It is estimated that 40% have suffered some falls, which restricts their daily activities [8,9]. Additionally, decreases in motor skills produce other limitations such as dependence, loss of autonomy and freedom to depend on a third party, decreasing the perception of well-being, quality of life and life satisfaction [10,11].

Additionally, falls are worrisome because they correspond to the main cause of injury and death in older adults, constituting a problem for public health worldwide, being the second leading cause of unintentional trauma deaths worldwide, with high associated costs per person in each trauma, which can exceed USD 3600 in developed countries [12]. It should be noted that half of older adults who have fallen are left with injuries that prevent them from getting back up [8], with consequences including pressure ulcers, osteopenia, loss of muscle mass and the subsequent functional impact, dehydration, hypothermia, pneumonia, and death [13]. In Chile, the annual prevalence of falls in older people is 35.3%, with 75% of them at home [9]. Thus, it is necessary to adapt the living spaces of the elderly.

One of the initiatives of the World Health Organization (WHO) is the strategy called the Decade of Healthy Ageing (2020–2030) [8], which aims for cities to meet the needs of the aging population to create friendly physical and social environments [14]. In this sense, studies confirm that these environments promote more active states in people, which reverts to improved quality of life [15]. This complements the active aging approach declared by the WHO more than two decades ago, understood as the process of making the most of opportunities to have physical, mental and social well-being throughout life, promoting extending the quality and life expectancy as long as possible [16]. It should be noted that health is a state of complete physical, mental, and social well-being, and not only the absence of affections or diseases [17]. In this sense, well-being is associated with quality of life—conditions promoting a perception of good living [18].

In this way, promoting improvements in the health of the elderly and maintaining their well-being is an investment in the health of the country because each of the older adults involves family networks that are affected when there is deterioration. The population of self-reliant older adults needs to be protected so that their comfort status is prolonged as long as possible. In this sense, the current advance in the technologies of information and the lowering of the costs of hardware allows us to face the problem of the elderly from a preventive and non-reactive perspective, that is, before the consequences on the health are irremediable. With this approach, smart homes based on Ambient Assisted Living (AAL) emerge, defined as environments that, using technology, aid people during daily activities [19], allowing them to respond to their behaviors in a non-intrusive or invasive way. The main functions that these smart homes fulfill are to serve users with physical disabilities; support people with visual impairment and/or deafness; monitor physiological signs with therapeutic devices; apply assisted therapy, intelligent device management, and smart home equipment; deliver comfort with leisure equipment, interactive communication, and physical activity systems.

The present paper describes the results of the implementation of the Quida platform presenting the findings and the impact evidenced on the quality of life of the study participants. Through a randomized controlled clinical trial, the validation of a cost/effective technological assistance platform was carried out that, in an intelligent environment implemented in the home of the elderly, monitors and accompanies the basic and instrumental activities of daily life to detect risk events and improve the quality of life. The platform, called Quida, consists of sensors to alert about abnormal levels of carbon monoxide, humidity, and temperature and detect falls and evaluate nocturia. 

On the other hand, temperature, light and movement sensors make it possible to monitor a person’s activity through actimetry (or actigraphy), which corresponds to the measurement of a person’s physical activity levels through the detection of body movement, allowing to detect activity or rest [20]. A randomized controlled clinical trial was carried out, whose objective was to determine the impact in terms of the quality of life of a non-intrusive sensor platform among independent older adults living in the community. This paper shows the results obtained from the study. Although some studies point to difficulties in monitoring users at home, Quida demonstrates that it is possible to detect presence and movement to identify behavior patterns in the sample studied at different time intervals, which is useful to support their medical evaluation. It begins by describing the current approach in assisted environments for older adults. Next, the sample and the study methodology are characterized, presenting in an image the action protocol for monitoring Quida as well as the location and functions of the sensors installed in the home. After the results, the discussion and conclusion are addressed.

## 2. Use of Assisted Environments for Older Adults

Demographic changes show that the aging population demands solutions from different approaches, where advances in technology for assisted environments in homes for the elderly become a way with achievements that demonstrate satisfactory progress. In this sense, it should be noted that the true need for care is dimensioned when it is understood that this population is a diverse group, which presents different expectations, that they are aware of their rights and are active participants in the construction of health [21]. Accordingly, information and communication technologies contribute to the empowerment and self-care of the elderly, maintaining their autonomy in decision-making. However, their use is conditioned by commitment and involvement in the design and adaptation of environments, which implies considering user preferences, simplicity, comprehensibility, perceived usefulness and ease of use [22].

One of the most important objectives of health research is the improvement of the living conditions and well-being of the elderly, especially those who live alone, since they may experience unwanted or dangerous situations in their daily life at home due to physical, sensory or cognitive limitations, such as forgetting their medication or bad eating habits [23]. In this line of studies, sensor-based recognition of human activity has been a widely addressed domain, with systems learning from a set of training samples to classify actions into a predefined set of actual field activities. We know that human behavior is variable; therefore, a recognition system should be able to learn and adapt continuously while retaining knowledge of learning activities or highlighting novel behavior, which could be potentially risky [24].

In the face of an aging world population, coupled with the pandemic events they have faced, sensor-based home care systems for monitoring older people seem to provide an effective solution. Evidence indicates that they are most useful when implemented non-intrusively through different visual and audio sensors, being ideal, in this case, the techniques of Artificial Intelligence and Computer Vision [25]. One aspect mentioned by the authors is that most of the studies on smart homes or health monitoring were tested in laboratory settings and were proofs of concept [26]. However, unintentional falls in older adults are a high-risk area, and their incidence imposes a significant economic burden on the health system [27]. Therefore, portable inertial sensors have grown in popularity as a means to objectively assess the risk of falls [28], generating studies aimed at its validation as non-intrusive systems for controlled environments to detect falls and other events and that do not compromise the privacy of users [29,30,31,32,33,34,35,36], being also relevant to obtain evidence of the impact on the quality of life of this age group.

## 3. Studies with Sensors in Older Adults

Aging in older adults is associated with an increased need for long-term care because mobility is lost, frailty increases and sequelae due to physical or mental illnesses increase disability rates [37]. Several studies suggest that environmentally assisted living (AAL) is a key enabler for an aging society [38,39,40,41], especially considering that providing protection and care for older adults living alone can involve high costs. In this regard, the World Health Organization points out that the global cost of caring for elderly people with dementia was US $818 billion in 2015, equivalent to 1.1% of the global domestic product (GDP), and the figure is estimated to continue to rise [42].

The AAL deals with the integration of sensors and smart devices that can be portable and environmental, which send information to process and store data to analyze physical and comfort parameters to allow adjustment according to the conditions of the older adult. The relevance of these systems lies in the fact that they deal with the prevention and care of older people both at home and outside the home in emergency situations [43]. Likewise, the technology to control sensors and devices aimed at providing comfort and safety to people in the living space has the ability to understand or learn the activities of its occupants [44]. These environments can be defined as products and services aimed at building intelligent environments for the benefit of older adults [45], which are made up of algorithms (software) and sensors (tools). The former is responsible for processing information [46], sensors as they can be non-portable and portable. Non-portable sensors allow for environmental data to be captured, namely motion (PIR, active infrared, ultrasound) or object information (RFID, pressure, and magnetic switches). Wearable sensors are used directly by the person. They are used to collect data on various aspects, including the accelerometer, gyroscope, glucometer, blood pressure, CO_2_, ECG, EEG, muscle activity, eye movement, blood oxygen saturation, perspiration or body temperature [46].

Some studies that have observed the behavior of older adults with sensors in a controlled environment (home) seek to determine its impact on well-being. The results point to the validation of the proposed models based on movement within the home and the heterogeneous use of objects [47]. To monitor activity and detect deviations from the long-term activity routine of elderly people living alone, researchers used low-cost, discrete binary sensors based on PIR detectors and magnetic door contacts [48]. However, one aspect highlighted by the authors is that a system designed to be used by real users faces many situations that cannot be controlled by the developers of the system and, therefore, be a source of errors [49]. For example, in falls [50], he notes that the algorithms tested using real-world fall data were much smaller than what was tested in a simulated environment, which definitely indicates that it is important to evaluate the proposed fall detection system in real-world conditions. In this regard, it is observed that despite the remarkable results recorded in the laboratory and reported in the literature, there are still very few real-life applications of the recognition of activities to support the independent living of the elderly [48].

## 4. Materials and Methods

The present study describes the results of the implementation of the Quida platform presenting the findings and the impact on the study participants’ quality of life. The research included a randomized controlled clinical trial (RCT) with registration NCT03891771, whose protocol was approved by the Scientific Ethics Committee of the Eastern Metropolitan Health Service and whose participants voluntarily signed the Informed Consent.

In this randomized trial, elderly participants (>65 years of age) perceived to be at an increased socioeconomic risk who lived alone in the city of Valparaiso or San Antonio and had previously obtained an economic subsidy granted by the Ministry of Housing and Urbanism in Chile were randomly allocated to receive one of two interventional strategies. The interventional arm received a complex sensor platform named Quida, which is briefly described below. Those allocated to the control arm received the usual care, with standard follow-up procedures as established by their primary care facilities. Participants with a history of dementia, substance or alcohol abuse, terminal illnesses (i.e., a life expectancy of fewer than six months), who owned pets within the residence and had a disability that rendered them unable to answer the questionnaire and those who refused participation were excluded from the trial. Randomization was handled by an independent statistician using a permuted blocks approach. Allocation sequences were kept concealed from other researchers for the trial duration.

All participants underwent an initial visit and were evaluated by professionals (medical doctors, physiotherapists and psychologists) with instruments designed to obtain a basic clinical profile and certify compliance with the selection criteria. 

**Figure 1:**
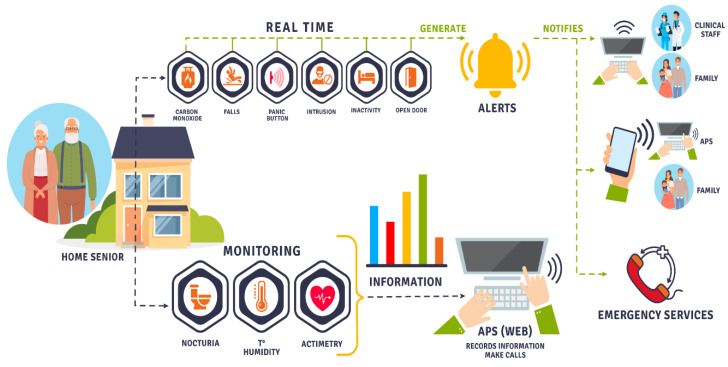
Quida: Integrated monitoring model.

The developed product, Quida, is the result of a process of continuous improvement through various research studies [29,30,31,32,33,34,35,36]. In particular, [29] used very low-resolution thermal sensors to classify falls and then alert care personnel. In addition, the performance of three recurrent neural networks for detecting falls was analyzed: short-term memory (LSTM), closed recurrent unit and Bi-LSTM, following, like many learning algorithms, a training phase with different test subjects. After several tests, it can be noted that the Bi-LSTM approach surpasses the other techniques reaching 93% accuracy in fall detection. The bidirectional form of the Bi-LSTM algorithm is estimated to give excellent results because the use of its data is influenced by old and new information, which is compared with LSTM and GRU. The information obtained through this system does not compromise the privacy of the user, which constitutes an additional advantage of this alternative. Quida consists of an integrated monitoring system that is continuous and non-intrusive to the daily activities of older people and capable of detecting risk events or accidents inside and outside the home without using devices that adhere to the body or monitoring private activities without authorization. When a possible event is detected (gas leak, fall or action of the emergency button), an alert is generated that is sent to the relatives and a telephone center (health or community center). If a letter is generated, the protocol is as follows (Figure 1): The staff of the telephone exchange calls the patient by phone to ask him how he is. In option A, if the patient responds and is not facing an emergency, this false positive is recorded on the platform. Otherwise, if the patient has an emergency, they call the emergency services or make a clinic visit to later register on the platform. In option B, if the patient does not respond, her contacts are called, and if assistance is required, the emergency services are called, or a clinic visit is made. Emergency services may include police, fire, and health services, depending on the event occurring.

**Figure 2:**
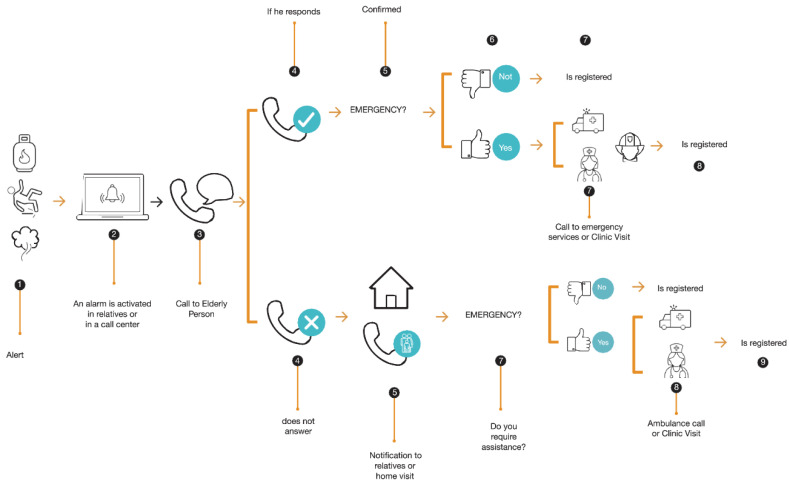
Quida alert system action protocol.

Thus, the Figure 2 shows the Quida alert system action protocol. When a possible gas event, fall or emergency button is detected, an alert is generated, sent to family members and a telephone exchange. This telephone exchange is responsible for calling the patient by phone to ask how he is. If the patient responds and is not facing an emergency, this false positive is recorded on the platform. Otherwise, the emergency services are called if the patient has an emergency, or a clinical visit is made to later register it on the platform. In the event that the patient does not respond, the patient’s contacts are called, and in case of requiring assistance, the emergency services are called, or a clinical visit is made and subsequently registered on the platform.

**Figure 3:**
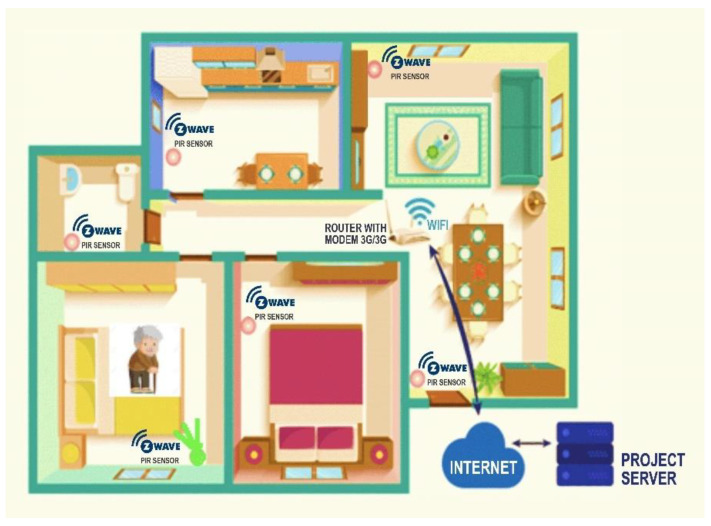
Detection system of basic and instrumental activities of daily living.

Quida is presented as a new model of care for the elderly, integrating support and health care networks with a technological monitoring system that allows for continuous accompaniment and protects the elderly. This system is proposed as a way to study human behavior by capturing, analyzing and permanently monitoring basic and instrumental activities in the daily life of older people (Figure 3).

The purpose of the Quida System is to detect events of risk to physical integrity by analyzing the environment and detecting changes in basic behavioral patterns such as sleep habits, night activity, and a sedentary lifestyle, among others. To do this, non-intrusive sensors were used, which do not violate the privacy of people, focusing solely on improving well-being and quality of life, and reducing direct supervision by family members and caregivers.

Quida is composed of a network of sensors and is oriented to:

1. Monitoring of environmental variables: MQ-9 sensor for gas detection. This sensor detects gas concentrations from 100 ppm to 10,000 ppm. DHT11 sensor to measure humidity and temperature. This sensor measures a temperature range from 0 °C to 50 °C with an accuracy of 0.2 °C and 20% to 90% relative humidity with 5% accuracy.
2. Accident monitoring: Raspberry microcontroller with MLX90640 thermal sensor for fall detection. The sensor is a 32 × 24 pixel thermal infrared array that has a temperature range of −40 °C to 85 °C. The values are transmitted via I2C and received by the raspberry to be processed.
3. Activity monitoring: To assess actimetry (or actigraphy) within the home. We used the Aeotec ZWA005 Trisensor, which has the ability to detect the presence and displacement of a person in a room through the measurement of movement, temperature and light. This sensor measures a temperature range of −15 to 50 °C with 1 °C accuracy, from 0 to 22,595 lux with 30 lux accuracy and a maximum of 7 m of motion sensitivity. It is also possible to monitor activity using the fall sensor (Raspberry + MLX90640), detecting presence and movement through heat variation in the room. The system also makes it possible to evaluate nocturia. For this, conductivity electrodes were used, which correspond to steel plates installed in the toilet and allow for the detection of urination events through the change in conductivity in the water.

The trial’s primary endpoint was health-related quality of life scores using the EuroQOL-5D questionnaire measured one month after randomization. This EuroQol-5D (EQ-5D) is a standardized questionnaire that quantifies health-related quality of life. It is divided into two sections, a descriptive section and a visual analog scale (VAS), at the end of the questionnaire. The descriptive system comprises five dimensions: mobility, self-care, usual activities, pain/discomfort and anxiety/depression. The EQ-VAS records the patient’s self-rated health on a vertical visual analog scale. Results are presented as a descriptive profile or as an index value calculated from the descriptive component, which is country-specific. Visual analog values can range from 0 to 100, with higher values indicating an overall better health-related quality of life. The EQ-5D tool was chosen because of its extensive validation across a wide range of settings to assess the quality of life, its simplicity of use and its availability in the Spanish language [51,52,53,54]. This questionnaire has also been validated among community residents in Chile [55]. These properties have been highlighted in the manuscript. Outcome assessors, statisticians and investigators were kept unaware of treatment allocation. All analyses were carried out under the intention-to-treat principle. 

## 5. Results

A total of 69 participants were randomized, with 32 allocated to the control arm and 37 to the interventional arm. Most were female (55 participants, 79.7%) with a mean age of 69.4 ± 7.6 years. The most common comorbidities were arterial hypertension (50 participants, 72.5%) and diabetes mellitus (24 participants, 34.8%). Seven participants (10.1%) had previously received a diagnosis of depression. Overall, participant characteristics were well-balanced between study groups, with one exception. Patients allocated to the control arm showed significantly higher baseline health-related quality of life scores as measured by the EQ-5D (0.798, IQR 0.694–1.00 vs. 0.59, IQR 0.456–0.798 points, *p* < 0.01). A complete summary of participant characteristics is presented in Table 1.

### 5.1. Actimetry

Actimetry is a method that allows the monitoring of activity in a non-invasive way through the detection of movement, the hypothesis of the study was that, through the implementation of thermal sensors and Tri Sensor (movement, temperature, light), it is possible to analyze the gradients of the pixels and the mobility of the person, to classify the basic and instrumental activities of the daily life of older adults with an estimated accuracy greater than 85%. To test this hypothesis, sensor networks were installed in different rooms of the homes of the participating older adults and the captured data were analyzed. The distribution of the sensors in the homes was as follows: (i) a raspberry microcontroller with thermal sensor (MLX90640) in the living room and in the master bedroom; (ii) a tri-sensor in the dining room and in the secondary bedroom; (iii) a temperature measurement sensor and a gas detection sensor in the kitchen. 

**Figure 4:**
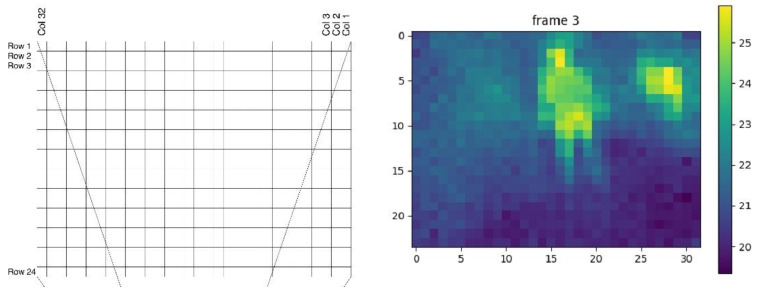
Representation of an image provided by the thermal sensor.

The system’s sensor network can detect presence and movement. The Tri sensors detect presence through increased temperature and movement through the PIR sensor, which detects movement by rapid changes in infrared energy, sending a signal when the person passes through the sensor. In the case of the raspberry, the low-cost MLX90640 thermal sensor composed of 768 pixels (32 × 24 pixels) allows for detection presence, movement and location (Figure 4).

In Figure 4, the image on the left shows the 32 × 24 pixel array of the MLX90640 sensor; the data is received as floating numbers corresponding to the temperature of each pixel. The image on the right shows the arrangement processed in color. In this way, the data from the presence sensor yield results through temperature arrangements, indicating the room of the older adult and if he was for a certain period. To obtain these results, an activity measurement algorithm was developed that compares the temperature of each pixel of the array with the background temperature used as a reference, calculated with a sliding window. If this difference is greater than a set threshold, a presence is detected. Thus, using algorithms, presence tracking allows you to calculate an activity rate for each hour.

**Figure 5:**
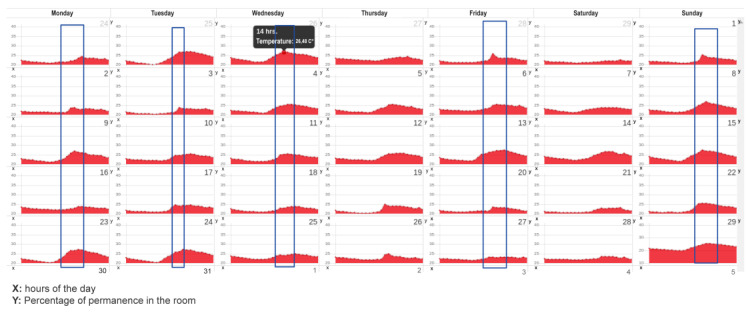
Activity detection representation.

The data received from the sensors are stored in a database and consist of the person’s RUT (Single National Role), movement, temperature, room brightness, and registration date. As a result, maps and tables of activities of each participant are obtained with sensors installed in their home. These tables and maps can be viewed on the platform and highlight the places where people stay the longest. In addition, it is possible to follow this parameter in two pieces of the same house. It is also possible to calculate the average activity corresponding to the average of one month of activity for each hour of the day. This activity corresponds to the average proportion of time, in relation to an hour in which a person remained in a room where the sensors were installed, allowing to outline trends or patterns of behavior such as hours of getting up and going to bed and peaks of activity. Figure 5 shows an example of the latter in a house for eight months.

Figure 5 shows that through the temperature sensor in the kitchen, it is possible to deduce a pattern of behavior. Between 13:00 and 14:00, the older adult is in the kitchen.

### 5.2. Impact on Behavior Pattern

#### 5.2.1. Positioning and Trajectories

**Figure 6:**
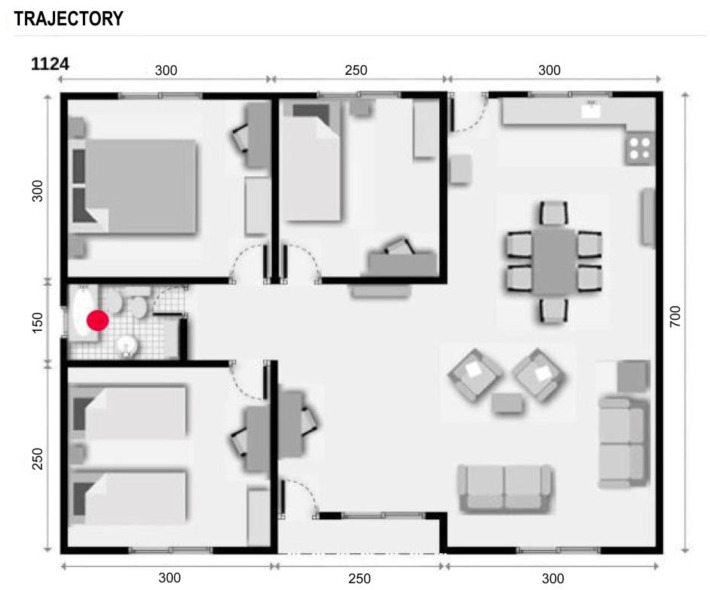
Representation of the location of the older adult at a given time.

Through the detection of presence, it was possible to identify the location of the elderly within their home and through the detection of the movement of the trajectories they made during the day, allowing them to identify patterns of daily behavior (or routines). A diagram was developed to visualize their location and corroborate the operation of the sensors (Figure 6).

To identify if the older adult was performing an activity inside a room, the 32 × 24 pixel arrangement of the thermal sensor was analyzed so that the difference between the pixels delivered activity information. For example: Watching television: Presence detected in the living room, low variation of the pixel matrix of the thermal sensor or low variation of the sensor that detects movement. In this way, it was possible to detect abnormal inactivity and send an alert through the system.

#### 5.2.2. Night Activity

**Figure 7:**
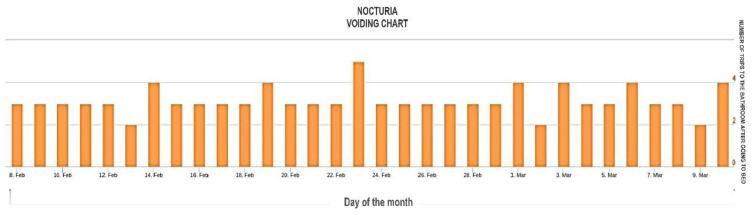
Daily nocturnal urination events.

To evaluate the nocturnal activity, the record of presence in the rooms of the home and the trajectories made during the night were analyzed, linking them with the nocturia sensor, which records urination events. The high frequency of nocturnal urination events (Figure 7), apart from causing sleep problems, can be symptoms of diseases such as diabetes and kidney problems. Thus, their early identification alerts the clinical team of possible complications in the patient to take action in a timely manner.

The x-axis is the day of the month. The y-axis is the number of times the person went to the bathroom after going to bed. These data were entered manually by each older adult in a card that was later entered into the system. The graphs were repeated; therefore, only the bar graph was left. On the other hand, if the nocturnal activity is not related to urination events, the system contrasts the usual behavior pattern with the current one, alerting of possible anomalous nocturnal behaviors and if these are being prolonged over time, which negatively impacts the quality of life of the elderly.

### 5.3. Impact of the Intervention on Quality of Life

**Figure 8:**
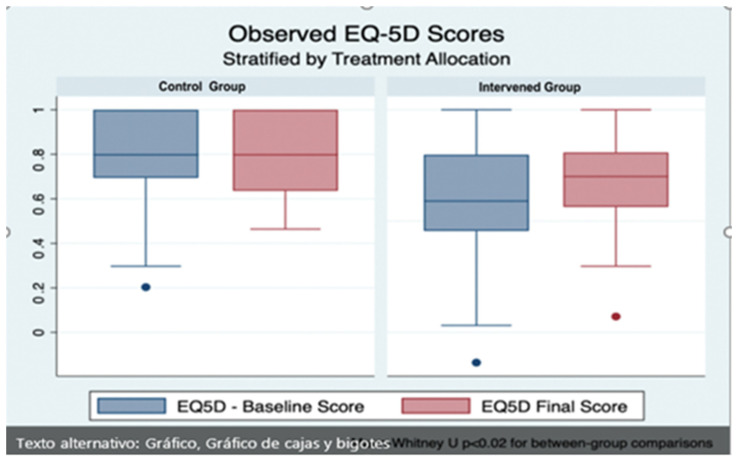
Boxplot showing the overall effects of the Quida platform in EQ-5D questionnaire between groups, before (blue) and after (red) the intervention. A statistically significant improvement from the baseline score was noted amongst participants allocated to the interventional arm (*p* < 0.01 Wilcoxon sign-rank test). When these changes were compared between groups, statistical significance was reached (Mann–Whitney U-test *p* < 0.03).

After completing the follow-up, the median score in the EQ-5D was 0.80 (IQR 0.64–1.0) in the control arm and 0.70 (IQR 0.56–0.81) in the control group. When comparing these scores, no evidence of a statistically significant change was found between groups (*p* = 0.23, Mann–Whitney U-test). However, an additional comparison was carried out to take the baseline imbalance in EQ5D scores between the groups that were mentioned above. The control arm showed no change in its quality of life measured by EQ-5D, with a median change of 0 points (interquartile range, IQR -0.163 to 0.202, intra-group *p*-value 0.77, Wilcoxon sign-rank test). On the other hand, the intervention arm showed a median increase in its EQ-5D score of 0.135 points (IQR 0–0.302 points, intra-group *p* < 0.01 Wilcoxon sign-rank test), which represented a 22% increase from baseline. When these differences from baseline between groups were compared using the Mann–Whitney U-test, statistical significance was reached (*p* < 0.03). The observed difference between groups is shown in Figure 8.

## 6. Discussion

Population aging is a worldwide phenomenon. Technology can help improve the quality of life for the elderly by improving their living conditions, providing support in their environment and fostering their independence. In this study, an assisted living environment using unobtrusive sensors significantly increased overall quality of life as measured by the EQ-5D score. However, this increase was only apparent after adjustments for baseline imbalances in this measure were made. The reasons behind this improvement are likely multifactorial. An assisted living platform with sensors aimed at monitoring environmental and individual variables could result in an enhanced perception of safety for the participant, which in turn might result in feelings of increased independence, engagement in activities of daily living and mobility. Furthermore, the unobtrusive nature of the platform results in minimal interference with everyday routines. Our findings are concordant with experiences from other trials, albeit data specifically pertaining to the quality of life is scarce. For instance, in 2015, Grant and coworkers reported significant increases in patient satisfaction among elderly participants allocated to a platform that included Telehealth sensors [56]. In another experience, Torkamani et al. also reported an improvement in quality-of-life measurements among caretakers of elderly persons with dementia that used a platform including devices to permit remote monitoring [57]. 

Previous studies focused on the analysis of human activity could not detect the interior location of the user [58,59,60]. The study with Quida showed that it is possible to detect the presence and movement of people to identify patterns of behavior in the sample studied. In addition, before the COVID-19 pandemic, older people were somewhat reluctant to use technology, which could be seen as a constraint to the development and implementation of this type of intervention. It should be noted that the sample of this research was not faced with manipulating devices or carrying any element or measurement technology in its body. However, currently (post-pandemic), the benefits of new technologies seem to be more accepted than before by this age range [26], which presages a future more auspicious for use. The degree of acceptance and benefit perceived by older people with the use of monitored environments is evidenced in the modification of the indices of quality of life reflected with a difference in favor when comparing the measures before and after the intervention. However, scientific evidence points out that about 56% of the studies on smart homes and home health monitoring technologies were carried out or tested in laboratory settings and were proof of concepts [26]. In this case, the Quida System was tested in the home of the participants with continued use for 26 months, 24 h a day.

The strengths of this study include its randomized design, the triple-masking of groups (participants, clinical outcome evaluators and analysts), the lack of losses of follow-up and the use of well-validated clinical scales to assess complex endpoints, such as quality of life. It also has important limitations. One of the most relevant ones is the baseline imbalance that was found between participants at baseline, with an overall higher EQ-5D score present in the control arm. This made detecting potential changes in quality of life more difficult to discern. We approached this problem by comparing the overall change from baseline between study groups, a method that seemed appropriate given our trial’s relatively small sample size. This small sample size represents a second key limitation. With a greater number of participants, it would also have been possible to conduct multivariable analyses to take baseline EQ-5D scores into account, and additional comparisons to further elucidate differential effects of the platform in participant subgroups could have been made. A third consideration stems from the characteristics of included participants. We solely evaluated independent elderly persons that lived alone and had no pets to assess our platform, a situation that might not represent the majority of the elderly population. These limitations need to be kept in mind when considering our results.

In addition, two key challenges were detected. First, when using non-intrusive or portable sensors, their accuracy has environmental restrictions. Visitors and pets of other residents hamper the reliability of the measurements since they might not represent the subject of interest. Other challenges include those arising from confidentiality concerns regarding the handling of sensitive data. It is necessary to ensure privacy protection at all times, given the sensitive nature of in-home monitoring data.

## 7. Conclusions

An approach based on the presence and movement detection has been presented to identify patterns of behavior in older people within the home. The algorithms were developed to allow the visualization of adult behavior at different time intervals to support their medical evaluation. In addition, an approach using datasets from real patients was evaluated. The results indicate that presence scores may illustrate the evidence of patient follow-up. In addition, it is possible to check when the patient has abnormalities in their daily activities (for example, family visits). Based on the above, living in more protected environments and homes is a part of what can be achieved today, providing timely assistance through non-intrusive monitoring to the elderly. The challenge now is for state standards for housing design and construction to incorporate evidence of technological advances because safe environments promote a better perception of quality of life. To advance this research topic in Chile, the team plans to increase the number of patients and the duration of measurements for the dataset to broaden their knowledge about monitoring patients within their own homes.

The futresearch team’s future work focused on how to complement the intelligent environment thailable today with three or four monitoring modules. New monitoring modules need to be integrated, for example, urine analysis, to detect some risk variables. A second challenge is to improve what has been achieved so far, which is linked to four variables: one is to reduce the size of the sensors to look less invasive—that is, they are less noticeable in the environment; reduce the associated cost; improve connectivity and improve information analysis. There are places with very poor connectivity that creates data blindspots. Improvement is required in terms of the sensitivity and specificity in the system related to how improvements in the artificial intelligence algorithms used to detect falls, for example. Finally, the analysis of the historical data to investigate patterns of behavior needs further improvement.

## Figures and Tables

**Table 1:** Characteristics of the study sample.

Characteristic	Control Group (n = 32)	Intervention Group (n = 37)	Total (n = 69)	*p*-Value
Clinical Characteristics				
Mean age (years) (SD)	67.8 ± 8.5	70.8 ± 6.5	69.4 ± 7.6	0.11 ^1^
Female sex (n, %)	26 (81.3%)	29 (78.4%)	55 (79.7%)	>0.99 ^2^
Asthma (n, %)	2 (6.3%)	4 (10.8%)	6 (8.7%)	0.68 ^2^
Chronic obstructive pulmonary disease (n, %)	1 (3.1%)	1 (2.7%)	2 (2.9%)	>0.99 ^2^
Arterial hypertension (n, %)	20 (62.5%)	30 (81.1%)	50 (72.5%)	0.11 ^2^
Diabetes mellitus (n, %)	10 (31.3%)	14 (37.8%)	24 (34.8%)	0.62 ^2^
Depression (n, %)	3 (9.4%)	4 (10.8%)	7 (10.1%)	>0.99 ^2^
Hypothyroidism (n, %)	3 (9.4%)	6 (16.2%)	9 (13.0%)	0.49 ^2^
Heart failure (n, %)	3 (9.4%)	5 (13.5%)	8 (11.6%)	0.72 ^2^
Ischemic Stroke (n, %)	2 (6.3%)	1 (2.7%)	3 (4.4%)	0.59 ^2^
Osteoarthritis (n, %)	11 (34.4%)	16 (43.2%)	27 (39.1%)	0.47 ^2^
Clinical Evaluation Scales				
Median Minimental State Evaluation Score (IQR)	30 (27–30)	29 (27–30)	29 (27–30)	0.27 ^3^
Median Barthel Index Score (IQR)	100 (100)	100 (100)	100 (100)	0.98 ^3^
Median Lawton Index Score (IQR))	8 (8)	8 (8)	8 (8)	0.85 ^3^
Median EQ5D Baseline Score (IQR)	0.798 (0.694–1.0)	0.590 (0.456–0.798)	0.698 (0.590–0.800)	<0.01 ^3^

^1^ Student’s *t*-test. ^2^ Fisher’s Exact Test. ^3^ Mann–Whitney rank-sum test. SD—Standard Deviation. IQR—Interquartile range.

## Data Availability

The data and results are available in clinical trials with registration NCT03891771.
